# Muscle Coordination Control for an Asymmetrically Antagonistic-Driven Musculoskeletal Robot Using Attractor Selection

**DOI:** 10.1155/2018/9737418

**Published:** 2018-09-12

**Authors:** Shoichiro Ide, Atsushi Nishikawa

**Affiliations:** ^1^Interdisciplinary Graduate School of Science and Technology, Shinshu University, Ueda 386-8567, Japan; ^2^Faculty of Textile Science and Technology, Shinshu University, Ueda 386-8567, Japan; ^3^Division of Biological and Medical Fibers, Institute for Fiber Engineering (IFES), Interdisciplinary Cluster for Cutting Edge Research (ICCER), Shinshu University, Ueda 386-8567, Japan

## Abstract

Recently, numerous musculoskeletal robots have been developed to realize the flexibility and dexterity analogous to human beings and animals. However, because the arrangement of many actuators is complex, the design of the control system for the robot is difficult and challenging. We believe that control methods inspired by living things are important in the development of the control systems for musculoskeletal robots. In this study, we propose a muscle coordination control method using attractor selection, a biologically inspired search method, for an antagonistic-driven musculoskeletal robot in which various muscles (monoarticular muscles and a polyarticular muscle) are arranged asymmetrically. First, muscle coordination control models for the musculoskeletal robot are built using virtual antagonistic muscle structures with a virtually symmetric muscle arrangement. Next, the attractor selection is applied to the control model and subsequently applied to the previous control model without muscle coordination to compare the control model's performance. Finally, position control experiments are conducted, and the effectiveness of the proposed muscle coordination control and the virtual antagonistic muscle structure is evaluated.

## 1. Introduction

Human beings and animals move flexibly and dexterously by controlling their musculoskeletal system with the brain. To understand and imitate the flexible and dexterous motion of human beings and animals, several musculoskeletal robots have recently been developed. The primary driving mechanism in musculoskeletal robots is a tendon-driven assembly using motors [1, 2] or pneumatic artificial muscles (PAMs). In particular, using PAMs as actuators enables flexible movement of the musculoskeletal robot compared with conventional robots that use motors because the compressibility and low viscosity of air provide compliance and rapid contraction. The extremely high power-to-weight ratio of the PAM is also good for flexible and dynamic motion.

The musculoskeletal robot comprises antagonistic-driven systems. An antagonistic-driven system includes two or more actuators for joint movement. The actuators are antagonistically arranged around one link, and their output characteristics and arrangements are, for the most part, symmetrical.

Many studies have proposed the musculoskeletal robots comprising this simple antagonistic system, and various control methods (e.g., PID control, neural network, and fuzzy logic) have also been proposed for musculoskeletal robots [3–8]. However, the drive system of our musculoskeletal robot [9] differs from a simple antagonistic-driven system because the output characteristics and the arrangements of the actuators of the robot are not symmetrical.

This robot has two kinds of PAM actuators, a monoarticular muscle that drives one joint and a polyarticular muscle that drives multiple joints consecutively. Therefore, the mechanism that drives each joint is not symmetrical. Furthermore, the actuators are not arranged symmetrically, although each actuator is antagonistically arranged around each link. Since each actuator is not arranged symmetrically and the sensors for measuring each joint angle are not arranged, design of the control system for the robot is more difficult and challenging.

Honda et al. [9, 10] proposed a biologically inspired control method using muscle coordination for the robot. They hypothesized that human beings use the synergies between antagonistic muscle pairs when joints move, and they defined two parameters, the antagonistic muscle ratio and the antagonistic muscle activity, as the key parameters in human muscle coordination. The parameters are computed by a PID controller [10] and are implemented to control the angle of the robot's joints.

Although good control performance was obtained for one joint, the controller did not work well for multiple joints of the robot [9]. An adaptive method that dynamically and adaptively searches the parameters for muscle coordination was required because PAMs have time variance, compliance, high hysteresis, and nonlinearity. In general, this search problem can be formulated as a combinatorial optimization problem of minimizing an object function subject to the search variables, which requires a precise model (object function) in advance, but system identification against an asymmetrically antagonistic-driven PAM system having nonlinear dynamics is difficult.

As an alternative to such a model-based theoretical approach, we believe that heuristic control methods inspired from living things are important for the control of the musculoskeletal robot. The musculoskeletal structures of human beings and animals are antagonistic-driven systems. They are also asymmetrically antagonistic-driven systems because monoarticular muscles and polyarticular muscles are arranged around various joints. Human beings and animals move flexibly by controlling their various muscles dexterously.

Recent research about the mechanisms of living things indicates that a biological system behaves flexibly using noise [11]. *Escherichia coli* (*E. coli*) cells usually prefer to switch to an adaptive attractor using noise to survive better in a new external environment after the environmental conditions have been changed. This adaptive behavior of the *E. coli* cells is known as attractor selection [12]. The novel control method based on the attractor selection has been proposed and applied to a signal-control method for traffic networks [13], network management [14, 15], android motion generation [16], robot navigation and locomotion [17–19], robotic arm control [20–22], and endoscopic surgery [23].

**Figure 1:**
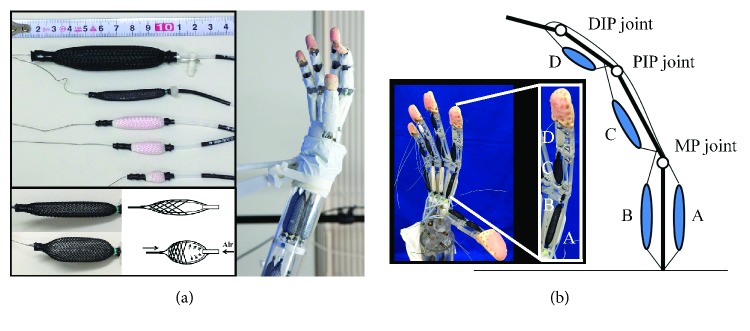
Control target and purpose. (a) Our five-fingered robot hand (right photograph) and pneumatic artificial muscles (PAMs) (left photographs) used as the actuator of the robot hand. These muscles are the McKibben actuator made in SQUSE Inc. and are driven by supplied compressed air (bottom-left pictures). (b) The structure of the index finger of the robot hand (the image and the closed photograph in the inset). The position of the tip of the index finger is controlled as a musculoskeletal robot. Various muscle lengths shown in (a) are arranged on the links of the index finger (they are also arranged for other fingers). The muscles A and B are of the same length. The muscle C is shorter than muscle A or B, and the muscle D is the shortest of all muscles on the finger. Each joint is driven from 0 deg (maximum extended state) to 90 deg (maximum flexed state).

The control method was described by a stochastic differential equation, input variables for the network systems or robots were computed by solving the equation, and the systems accomplished the tasks without the dynamics and model of the systems and environments. Since the attractor selection is conducted adaptively using noise in the systems or environments, the control method is robust for changes of tasks and environments. Attractor selection was applied to an asymmetrically antagonistic-driven musculoskeletal robot (Figure 1) with muscles arranged asymmetrically [24]. From the control experiment, the position of the tip of the robot was moved to the desired position by searching pressure for four PAMs individually using the attractor selection. The control time had to be more than 100 s to accomplish tasks. Therefore, modification of the control method is required to accomplish tasks quickly.

In this study, we propose a novel muscle coordination control method for the asymmetrically antagonistic-driven musculoskeletal robot using the attractor selection. The primary difference between the previous method [24] and the proposed one is that the proposed method introduced a virtual antagonistic muscle structure as a muscle coordination control model. Instead of individually and directly searching the PAM pressure in the actual asymmetric antagonistic muscle structure, the new method indirectly searches pressure for actual PAMs via a virtual symmetric antagonistic muscle structure.

First, muscle coordination control models of the musculoskeletal robot were built using virtual antagonistic muscle structures with a virtually symmetric arrangement of muscles. Next, the attractor selection was applied to the control model and also applied to the previous control model without the muscle coordination to compare control performance. Finally, position control experiments were conducted, and the effectiveness of the proposed muscle coordination control applied attractor selection and the virtual antagonistic muscle structure was evaluated.

## 2. Materials and Methods

### 2.1. Purpose: Control of the Position of an Asymmetrically Antagonistic-Driven Musculoskeletal Robot

In this study, a five-fingered robot hand (Figure 1(a)) inspired by the right hand of a human being was used, and the position of the tip of the index finger of the robot hand was controlled as a musculoskeletal robot.  Figure 1(b) shows the structure of the index finger. It has a three-degrees-of-freedom mechanism that is formed by four links (a distal phalange, an intermediate phalange, a proximal phalange, and a metacarpal) and three joints (distal interphalangeal (DIP), proximal interphalangeal (PIP), and metacarpophalangeal (MP)). Four PAMs are used: one actuator on the intermediate phalange, one on the proximal phalange, and two on the metacarpal for flexion and extension. The actuator for extension (referred to as the “extensor”) is a polyarticular muscle in which wire covers all joints. Therefore, the number of actuators for flexion and extension is not symmetrical.

Let *P*_e_, *P*_MPf_, *P*_PIPf_, and *P*_DIPf_ be the pressure supplied to the muscles A, B, C, and D, respectively, in Figure 1. The subscript “e” indicates the extensor, “MPf” indicates the flexor (the actuator for flexion) for driving the MP joint, “PIPf” indicates the flexor for driving the PIP joint, and “DIPf” indicates the flexor for driving the DIP joint, and they are calculated as follows:

(1) $$\begin{array}{c} P_{e}=P_{\max}\cdot x_{e}, \end{array}$$

(2) $$\begin{array}{c} P_{MPf}=P_{\max}\cdot x_{MPf}, \end{array}$$

(3) $$\begin{array}{c} P_{PIPf}=P_{\max}\cdot x_{PIPf}, \end{array}$$

(4) $$\begin{array}{c} P_{DIPf}=P_{\max}\cdot x_{DIPf}, \end{array}$$

where *x*_e_(∈[0, 1]), *x*_MPf_(∈[0, 1]), *x*_PIPf_(∈[0, 1]), and *x*_DIPf_(∈[0, 1]) are the normalized search variables and *P*_max_ is the maximum pressure supplied to the actuator. Since the actuators of the robot hand break if a pressure of more than 0.2 MPa is supplied, the value of *P*_max_ is set to 0.19 MPa.

### 2.2. Muscle Coordination Hypothesis

Honda et al. suggested the hypothesis that muscle coordination of human beings is a coordination of antagonistic muscles (the extensor and flexor) [9, 10]. They express the ratio of the coordination using two parameters, the antagonistic muscle ratio (Ar) and antagonistic muscle activity (Ac). The Ar is the value that regulates the ratio of pressure (*P*_e_ and *P*_f_) between the antagonistic muscle e (the extensor) and f (the flexor). Ar is calculated between 0 and 1 and is defined as follows:

(5) $$\begin{array}{c} Ar=\frac{P_{e}}{P_{e}+P_{f}}. \end{array}$$

Ac is the sum of pressure for the extensor e and the flexor f and is calculated by

(6) $$\begin{array}{c} Ac=P_{e}+P_{f}. \end{array}$$

In this study, Ac is always set to the maximum pressure for driving joints sufficiently. That is,

(7) $$\begin{array}{c} Ac=P_{\max}. \end{array}$$

Pressures *P*_e_ and *P*_f_ are calculated from (5), (6), and (7).

(8) $$\begin{array}{c} P_{e}=P_{\max}\cdot Ar, \end{array}$$

(9) $$\begin{array}{c} P_{f}=P_{\max}\cdot \left(1-Ar\right). \end{array}$$

**Figure 2:**
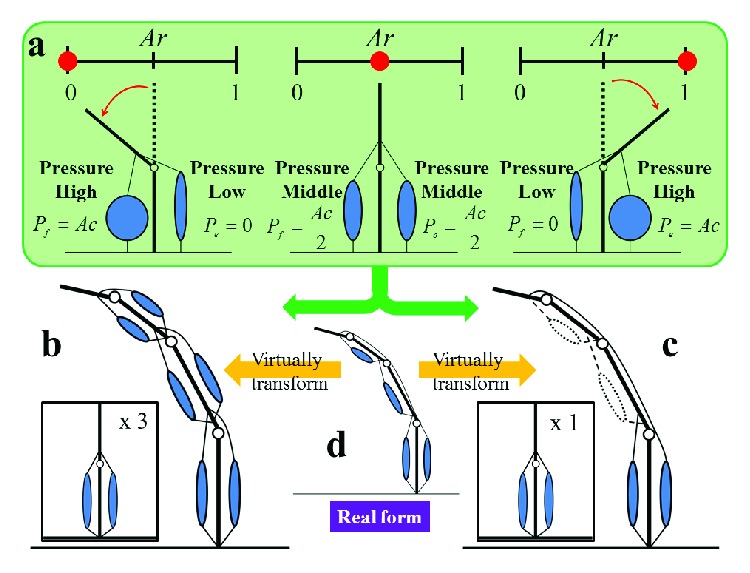
Muscle coordination and two types of virtual antagonistic muscle structures. (a) Principle of the muscle coordination using the antagonistic muscle ratio Ar and the antagonistic muscle activity Ac. When Ar is 0 (left picture), the pressure for the flexor is high (*P*_f_ = Ac) and the extensor is low (*P*_e_ = 0). When Ar is 0.5 (middle picture), the pressure for the flexor and the extensor is identical (*P*_f_ = *P*_e_ = Ac/2). When Ar is 1 (right picture), the pressure for the flexor is low (*P*_f_ = 0) and the extensor is high (*P*_e_ = Ac). Therefore, the pressures of the extensor and the flexor are determined by searching for the normalized variable Ar ∈ [0, 1]. (b) One of the virtual antagonistic structures. Two muscles are added virtually to the musculoskeletal system (shown in (d)) to form a symmetrically antagonistic arrangement on the intermediate phalange and the proximal phalange. Therefore, the virtual antagonistic structure has three simple antagonistic muscle structures (inset picture in (b)). (c) The other virtual antagonistic muscle structure. Two muscles on the intermediate phalange and the proximal phalange are decreased virtually from the musculoskeletal robot (shown in (d)), and the muscles are symmetrically arranged on the metacarpal. Therefore, the virtual antagonistic structure has one simple antagonistic muscle structure (inset picture in (c)). (d) The real form of the musculoskeletal robot that is controlled. The value of Ar (shown in (a)) is applied to the virtual antagonistic structure (shown in (b) or (c)) virtually transformed from the musculoskeletal robot (shown in (d)).

Equations (8) and (9) show that the pressures of the extensor and the flexor are determined by searching for the normalized variable Ar ∈ [0, 1].  Figure 2(a) shows the principle of muscle coordination using the antagonistic muscle ratio Ar and antagonistic muscle activity Ac.

### 2.3. Two Types of Virtual Antagonistic Muscle Structures

Four actuators were used in the musculoskeletal robot: the polyarticular muscle was used as the extensor and three monoarticular muscles were used as the flexor (Figure 1(b)). To calculate the pressure for each actuator based on the muscle coordination hypothesis, two methods that make the total number of extensors and flexors match virtually are proposed.

The first method is composed as follows. The virtual antagonistic muscle structure (Figure 2(b)) is composed so that the number of extensors is increased from one to three extensors. Next, Ar is applied to each antagonistic muscle to drive each joint. Hence, Ar is described as Ar_MP_, Ar_PIP_, and Ar_DIP_, and the pressures for the extensor and the flexor at each antagonistic muscle for driving each joint are denoted as *P*_MPe_, *P*_MPf_, *P*_PIPe_, *P*_PIPf_, *P*_DIPe_, and *P*_DIPf_. The pressures are calculated as follows:

(10) $$\begin{array}{c} P_{MPe}=P_{\max}\cdot {Ar}_{MP}, \end{array}$$

(11) $$\begin{array}{c} P_{PIPe}=P_{\max}\cdot {Ar}_{PIP}, \end{array}$$

(12) $$\begin{array}{c} P_{DIPe}=P_{\max}\cdot {Ar}_{DIP}, \end{array}$$

(13) $$\begin{array}{c} P_{MPf}=P_{\max}\cdot \left(1-{Ar}_{MP}\right), \end{array}$$

(14) $$\begin{array}{c} P_{PIPf}=P_{\max}\cdot \left(1-{Ar}_{PIP}\right), \end{array}$$

(15) $$\begin{array}{c} P_{DIPf}=P_{\max}\cdot \left(1-{Ar}_{DIP}\right). \end{array}$$

Finally, *P*_e_ that applies to the real extensor on the real form (Figure 2(d)) is calculated using

(16) $$\begin{array}{c} P_{e}=\frac{1}{3}\left(P_{MPe}+P_{PIPe}+P_{DIPe}\right). \end{array}$$

The second method is distinguishable in the following ways. First, the other virtual antagonistic muscle structure (Figure 2(c)) reduces the number of flexors to one. Next, Ar is applied to the virtual antagonistic muscle structure, and the pressures *P*_e_ for the virtual extensor and *P*_f_ for the virtual flexor are calculated using (8) and (9). Here, *P*_f_ is the total pressure for the three real flexors (*P*_MPf_, *P*_PIPf_, and *P*_DIPf_) and is defined as *P*_f_ ≤ *P*_max_. Finally, *P*_f_ is distributed to the three real flexors on the real form (Figure 2(d)). Two distribution ratios (Dr_MP_ and Dr_PIP_) are used, and the pressure for the three real flexors is calculated by

(17) $$\begin{array}{c} P_{MPf}=P_{f}\cdot {Dr}_{MP}, \end{array}$$

(18) $$\begin{array}{c} P_{PIPf}=\left(P_{f}-P_{MPf}\right)\cdot {Dr}_{PIP}, \end{array}$$

(19) $$\begin{array}{c} P_{DIPf}=P_{f}-\left(P_{MPf}+P_{PIPf}\right). \end{array}$$

Pressure for the real extensor is the same as that for the virtual extensor and is calculated using (8).

### 2.4. Attractor Selection Model

Let us consider the combinatorial optimization problem of minimizing an object function *U*(*x*) subject to the search variable *x* ∈ {*X*_1_, *X*_2_,…, *X*_*N*_}, where *X*_*i*_ is a feasible solution (an attractor) and *N* means the number of attractors. Rather than seek an optimal solution, we try to quickly find good approximate solutions. To accomplish this, our study uses the revised attractor selection model, which has been generalized as a stochastic differential equation [24]:

(20) $$\begin{array}{c} \frac{d}{dt}x\left(t\right)=f\left(x\left(t\right)\right)\cdot Activity\left(t\right)+\left(1-Activity\left(t\right)\right)\cdot \eta \left(t\right), \end{array}$$

where *t* is time, *x* is the search variable or state (∈[0, 1] for our case), the value Activity (∈[0, 1]) is the degree of accomplishment of the task, *η* is assumed as noise, and *f*(*x*) is the function that makes *x* converge to a suitable attractor. Typically, the function *f* can be represented as *f*(*x*) = −*∂U*(*x*)/*∂x* if the objective function *U*(*x*) is known precisely in advance.

This model searches for a solution (the attractor) that successfully accomplishes the task using noise, and the Activity makes the behavior of the total system change. Notice that as Activity increases, the term *f*(*x*) · Activity becomes more dominant in (20) and the state transition becomes more deterministic. Consequently, state *x* tends to be entrained into a suitable attractor, where it remains despite the persistent noise. By contrast, decreasing the Activity increases the dominance of the noise *η*, thereby flattening the potential landscape. In this scenario, the transition becomes more probabilistic, like a random walk, and *x* is driven away from the attractor.

The function *f*(*x*) can be designed freely, even if the objective function *U*(*x*) is unknown or not precisely described. Two elements are required. The value of *x* must converge to the attractors, and the *x* must remain at a suitable attractor. To satisfy these elements, *f*(*x*)is defined as follows:

(21) $$\begin{array}{c} f\left(x\right)\equiv \overset{N-1}{\underset{i=0}{\sum }}\frac{k_{d}^{2}}{{\left(X_{i}-x\right)}^{2}+k_{w}^{2}}\cdot \frac{X_{i}-x}{\left\|X_{i}-x\right\|}, \end{array}$$

where *X*_*i*_ is the *i*th attractor, *N* is the number of attractors, *k*_*d*_ is the power that attracts *x*, and *k*_*w*_ is the range in which the attractor's power *k*_*d*_ is effective.

### 2.5. Employing the Attractor Selection to Determine the Pressure Supplied to Each Actuator

The selection of the attractor is employed to determine the pressure supplied to each actuator. The three methods used to determine the pressure using attractor selection are presented as follows.

The first method uses the real musculoskeletal structure (Figure 2(d)) and directly calculates the four pressures by searching for the four variables (*x*_e_, *x*_MPf_, *x*_PIPf_, and *x*_DIPf_) using the attractor selection. We refer to this as a pressure search-type controller. The pressure supplied to each actuator is calculated by (1), (2), (3), and (4). This method is the same as reported in our previous method in [24].

The second method uses the first virtual antagonistic muscle structure (Figure 2(b)). It searches for the three ratios (Ar_MP_, Ar_PIP_, and Ar_DIP_) using the attractor selection and supplies the pressure to each actuator using (10), (11), (12), (13), (14), (15), and (16). We refer to this as an Ar search-type controller.

The third method uses the second virtual antagonistic muscle structure (Figure 2(c)). It searches for the Ar value and the two distribution ratios (Dr_MP_ and Dr_PIP_) using the attractor selection and then supplies the pressure to each actuator using (8), (9), (17), (18), and (19). This is referred to as an Ar and Dr search-type controller.

Notice that the search space for all the variables (*x*_e_, *x*_MPf_, *x*_PIPf_, and *x*_DIPf_; Ar_MP_, Ar_PIP_, and Ar_DIP_; and Ar, Dr_MP_, and Dr_PIP_) ranges from 0 to 1.

## 3. Experimental Procedures

**Figure 3:**
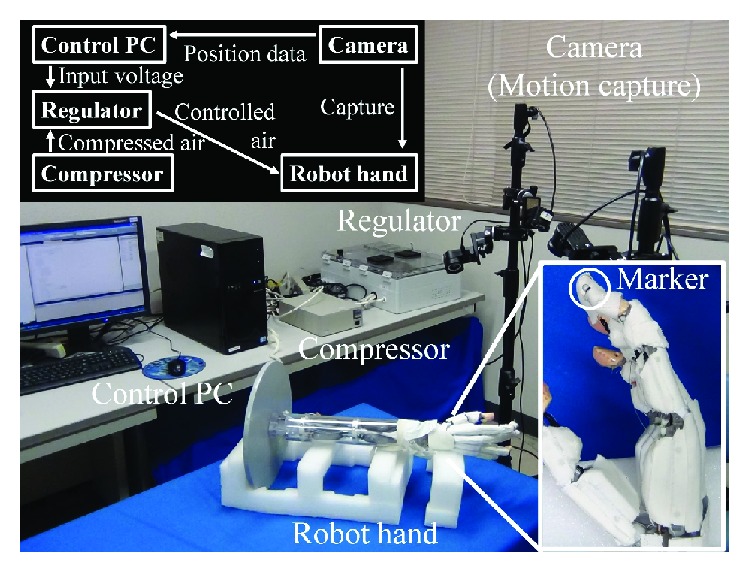
System overview. Two insets show the system flow (top-left) and the position of the marker captured by the cameras in an enlarged view of the robot hand (bottom-right). The marker is set to the tip of the musculoskeletal robot (the index finger). The input voltage from the control PC is converted to the controlled pressures by the regulators, and the musculoskeletal robot is driven by the pressures supplied to each muscle of the robot. The position of the tip is measured using the marker captured by the cameras and is saved to the PC.

A control experiment that makes the tip of the index finger of the musculoskeletal robot (see the bottom-right inset in Figure 3) move to the desired position was conducted using the above three controllers. Figure 3 depicts the experiment setup, which comprises the musculoskeletal robot (SQUSE hand G type, SQUSE Inc.), the control PC (MDV ADVANCE ST 6300B (MouseComputer Co. Ltd.), Windows XP, and an Intel Core i7 920 (2.67 GHz)), an A/D converter (AI-1664L-LPE, CONTEC Co. Ltd.), two D/A converters (AO-1616L-LPE, CONTEC Co. Ltd.), a digital output board (RRY-32-PE, CONTEC Co. Ltd.), a motion capture system (Nobby Tech. Ltd.), regulators (ITV0030, SMC Corporation), solenoid valves (S070-5DCO-32, SMC Corporation), and an air compressor (DPP-AYAD, Koganei Corporation). The sampling frequency was set at 100 Hz. The input signals were voltages generated by the control PC and were converted to pressures by the regulators. The output signals were the coordinates of the tip position [*E*_*X*_, *E*_*Y*_, *E*_*Z*_] sensed by the motion capture system. The Euclidean norm (the distance between the desired position [*E*_*Xd*_, *E*_*Yd*_, *E*_*Zd*_] and the tip position) was used as the evaluation index of the controller. The Euclidean norm is described by *l* and computed using

(22) $$\begin{array}{c} l=\sqrt{{\left(E_{Xd}-E_{X}\right)}^{2}+{\left(E_{Yd}-E_{Y}\right)}^{2}+{\left(E_{Zd}-E_{Z}\right)}^{2}}. \end{array}$$

And Activity of the attractor selection model is calculated from 0 to 1 using

(23) $$\begin{array}{c} Activity=-\frac{l}{l_{\max}}+1. \end{array}$$

Here, *l*_max_ is the maximum value of a norm computed from the desired position and the tip position on either the maximum flexion, which is a steady state in which maximum pressure (0.19 MPa) is supplied to all flexors, or the maximum extension, which is a steady state in which maximum pressure is supplied to only the extensor of the robot.

Each norm was obtained in advance, and the larger norm was selected as *l*_max_. Two tasks were conducted in the experiment. The flexion task involved making the robot flex to a desired position from an extended state, the extension task involved extending the robot to a desired position from a flexed state, and the tasks were changed after a constant time. First, the flexion task was conducted. After a constant time, the desired position was changed and the extension task was conducted. The control time was set at 60 s, and the task was changed 30 s after the control was started. The first position and the desired position were defined when the robot was in a steady state after constant pressure was applied to each actuator. Each position was captured in advance.

In this experiment, the pressure value, represented by *P*_e_, *P*_MPf_, *P*_PIPf_, and *P*_DIPf_ (0.05, 0.15, 0.05, and 0.05, resp.), was applied to each actuator to determine the desired position for the flexion task, and 0.15, 0.05, 0.05, and 0.05, respectively, were applied to each actuator to determine the desired position for the extension task. The initial values of *x*_e_, *x*_MPf_, *x*_PIPf_, and *x*_DIPf_; Ar_MP_, Ar_PIP_, and Ar_DIP_; and Ar, Dr_MP_, and Dr_PIP_ were set to 0.9, 0.1, 0.1, and 0.1; 0.9, 1.0, and 0.0; and 0.9, 1.0, and 0.0, respectively, and the noise *η* was generated between −10 and 10. The parameter values in (21) were set as follows: *N* = 11, *k*_*d*_ = 0.01, *k*_*w*_ = 0.01, and *X*_*i*_ = 0.1 × *i*.

## 4. Results and Discussion

**Figure 4:**
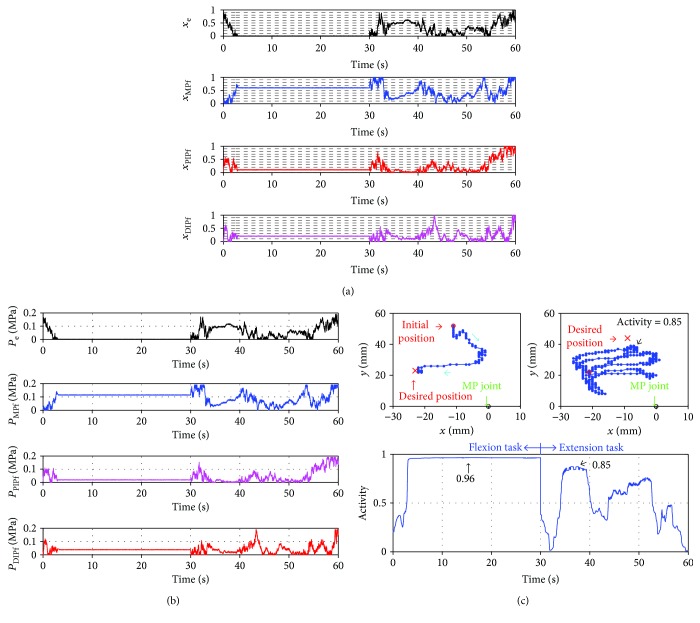
Results using the pressure search-type controller. (a) Plots of the transition of the search variables (*x*_e_, *x*_MPf_, *x*_PIPf_, and *x*_DIPf_) and the attractors (dashed lines). (b) Plots of the transition of the input pressures (*P*_e_, *P*_MPf_, *P*_PIPf_, and *P*_DIPf_). (c) Plots of the trajectory of the tip of the robot for the flexion task (top-left) and the extension task (top-right) and the transition of the Activity (bottom). Cyan arrows, green arrows, and black arrows show the direction of the movement, the MP joint position, and the value of Activity, respectively.

**Figure 5:**
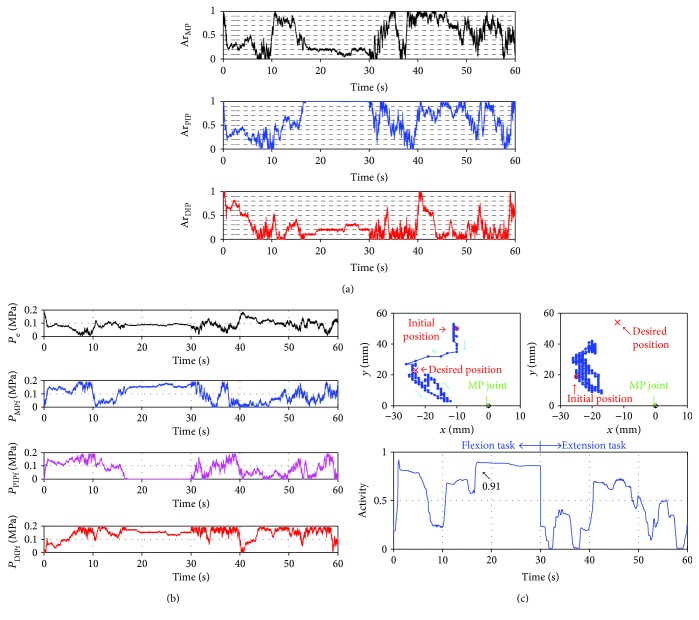
Results using the Ar search-type controller. (a) Plots of the transition of the search variable (Ar_MP_, Ar_PIP_, and Ar_DIP_) and the attractors (dashed lines). (b) Plots of the transition of the input pressures (*P*_e_, *P*_MPf_, *P*_PIPf_, and *P*_DIPf_). (c) Plots of the trajectory of the tip of the robot on the flexion task (top-left) and the extension task (top-right) and the transition of the Activity (bottom). Cyan arrows, green arrows, and black arrows show the direction of the movement, the MP joint position, and the value of Activity, respectively.

**Figure 6:**
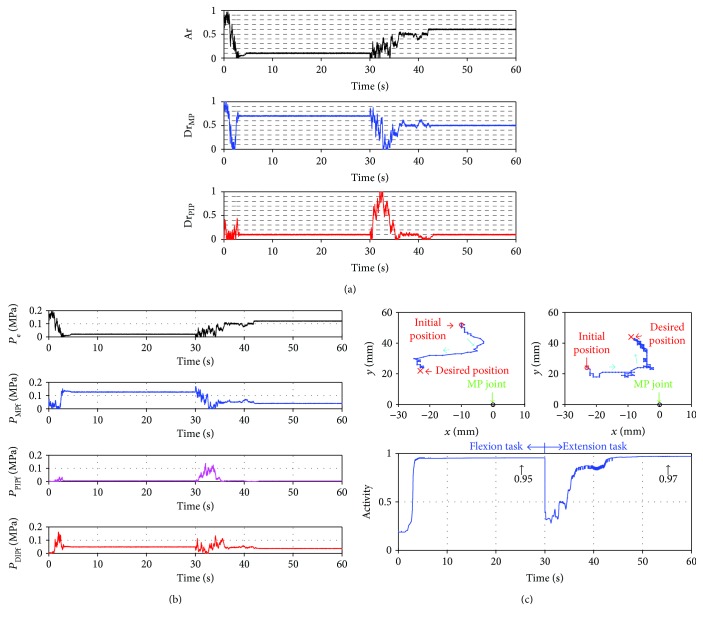
Results using the Ar and Dr search-type controller. (a) Plots of the transition of the search variable (Ar, Dr_MPf_, and Dr_PIPf_) and the attractors (dashed lines). (b) Plots of the transition of the input pressures (*P*_e_, *P*_MPf_, *P*_PIPf_, and *P*_DIPf_). (c) Plots of the trajectory of the tip of the robot on the flexion task (top-left) and the extension task (top-right) and the transition of the Activity (bottom). Cyan arrows, green arrows, and black arrows show the direction of the movement, the MP joint position, and the value of Activity, respectively.

Figures 4–6 show the results using the pressure search-type controller, the Ar search-type controller, and the Ar and Dr search-type controller, respectively. The transition of the search variables, the pressures supplied to each actuator, the tip position captured by the motion capture, and Activity of the attractor selection model were then plotted. In Figure 4, Activity increased from 3 s and became constant at 0.96. Therefore, the flexion task was almost accomplished, and the search variables converged to an attractor. However, the extension task was not completed. Activity increased from 33 s to 35 s and remained at nearly 0.8, but it decreased from 39 s and did not reach a high value. The difference in the accomplishment ratio of the task is caused by the relationship of pressure between the extensor and the flexor. When Activity increased and became constant in the flexion task, the pressure on the extensor (*P*_e_) decreased and the pressure on the flexor responsible for driving the MP joint (*P*_MPf_) increased. Therefore, the power for the flexion became large, and the robot flexed toward the desired position. In the extension task, pressures for the extensor (*P*_e_) and pressure for the flexor for driving the MP joint (*P*_MPf_) increased from 33 s to 35 s. Therefore, the power for the extension did not increase dramatically, and the robot did not accomplish the extension task. The difference between the pressure for the extensor and the flexor is important for achieving the task efficiently.

In Figure 5, Activity increased after 10 s and reached a constant value of 0.91 at 17 s. The flexion task was close to being accomplished, but compared with the pressure search-type controller, the accomplishment ratio was 5% lower and the control time for accomplishment of the task was 13 s longer. Therefore, the control was not as good as that of the pressure search-type controller. In the extension task, Activity did not attain a high value. Therefore, the extension task was not accomplished. As a result, the controller did not work well for the extension task. To efficiently accomplish the task, it was necessary that the differences between the pressures for the extensor and the flexors be easily calculated by the controller because the differences can decrease power which prevents accomplishment of tasks and can make the tip position of the robot move quickly to the desired position, but this controller cannot calculate the differences between the pressures as well as the pressure search-type controller. This controller searches for three Ar values independently. Therefore, each Ar takes on a different value, and the pressure for the flexor does not decrease satisfactorily. Thus, the power for flexion does not decrease sufficiently, and the extension task is not accomplished.

In Figure 6, Activity increased greatly and became constant at 0.95 for the flexion task. On the extension task, Activity increased gradually right after the change of the task and became constant at 0.97. Therefore, the flexion task and the extension task were accomplished. The Ar and Dr search-type controller is, thus, more highly adaptive for tasks than the pressure search-type controller and the Ar search-type controller. Especially, the effectiveness of the Ar and Dr search-type controller for the extension task was shown. The robot has four actuators arranged asymmetrically on the extension side and the flexion side. One actuator is installed on the extension side, and three actuators are installed on the flexion side. Therefore, the power for flexion easily becomes large compared with the power for extension. To make this robot extend sufficiently, the power for flexion must become small.

**Figure 7:**
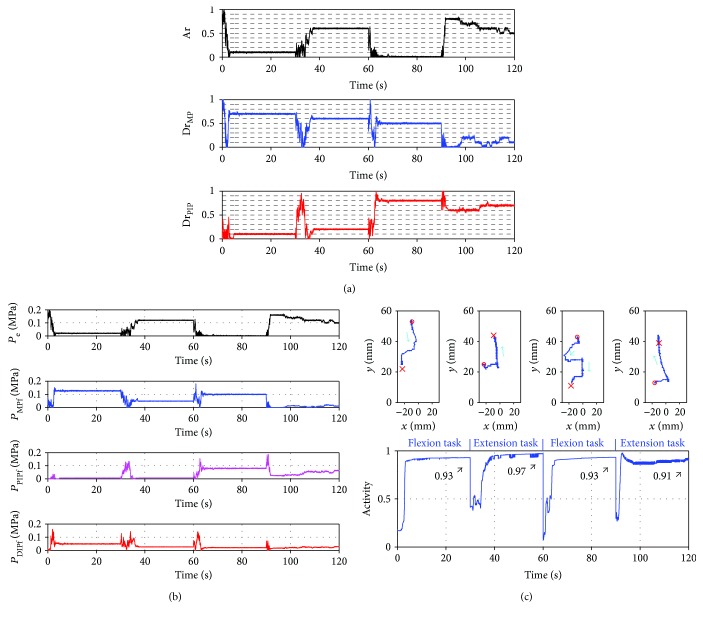
Results using the Ar and Dr search-type controller at four desired positions. (a) Plots of the transition of the search variable (Ar, Dr_MPf_, and Dr_PIPf_). (b) Plots of the transition of the input pressures (*P*_e_, *P*_MPf_, *P*_PIPf_, and *P*_DIPf_). In (c), the top images plot the trajectory of the tip of the robot: the first flexion task, the first extension task, the second flexion task, and the second extension task from the left image, whereas the bottom image plots the transition of the Activity. Cyan arrows and black arrows show the direction of the movement and the value of Activity, respectively.

In the Ar and Dr search-type controller, the difference between the power in the flexion and in the extension is determined easily because the difference between the pressures for the flexor and the extensor is calculated by searching only one Ar. Therefore, the power for flexion becomes small, and the robot can sufficiently extend. We also conducted the different task for the Ar and Dr search-type controller to show the adaptability of the controller.  Figure 7 shows the results of the experiment. We set four tasks, two flexion tasks and two extension tasks, which were conducted alternately. The desired position for each flexion task was different, and each desired position for each extension task was different. Each task was changed at the following constant times: 30 s, 60 s, and 90 s after the control is started. Experimental results show that Activity became the high value for each task. Therefore, the Ar and Dr search-type controller displayed good adaptability for the tasks in the experiments. Thus, it was shown that the proposed muscle coordination control of the Ar and Dr search-type controller using the attractor selection facilitated easy control of an asymmetrically antagonistic-driven musculoskeletal robot with a polyarticular muscle.

## 5. Conclusions

This work demonstrated a muscle coordination control of an asymmetrically antagonistic-driven musculoskeletal robot using attractor selection which is a biologically inspired search method.

First, muscle coordination control models of the musculoskeletal robot were built using virtual antagonistic muscle structures with a virtually symmetric arrangement of muscles, and the calculation methods of the input pressure for PAMs of the musculoskeletal robot with and without muscle coordination were shown. Next, the attractor selection was applied to both the muscle coordination control model and to another control model without the muscle coordination to compare the control performance. Finally, position control experiments were conducted, the effectiveness of the proposed muscle coordination control applied to the attractor selection was demonstrated, and it was also shown to be faster and more robust to accomplish the task by generating control commands virtually assuming a symmetrical and simpler metastructure rather than providing a control command according to the actual complex (asymmetric) antagonistic muscle structure.

Based on the virtual antagonistic muscle structure proposed in this research, we may be able to build a musculoskeletal robot that achieves a more complicated task faster by devising how to give noise [25] and adaptively updating the attractor structure [21]. In future work, the muscle coordination control method, using the attractor selection, will be applied to the multifingered robot hand formed by increasing the number of musculoskeletal robots (i.e., robot fingers). Furthermore, the effectiveness of the control method will be investigated for asymmetrically antagonistic-driven musculoskeletal robots, which have entirely different arrangements of muscles compared with our musculoskeletal robot.

The muscle coordination control method using attractor selection can be applied not only to musculoskeletal robots but also to human hands. Therefore, the control method will be applied to rehabilitation using functional electrical stimulations (FESs) [26, 27] as a novel approach to controlling human hands.

## Data Availability

The raw data for Figures 4–7 are available from the corresponding author upon request.
